# Susceptibility of *Caenorhabditis elegans* to *Burkholderia* Infection Depends on Prior Diet and Secreted Bacterial Attractants

**DOI:** 10.1371/journal.pone.0007961

**Published:** 2009-11-23

**Authors:** Vaughn S. Cooper, Wendy A. Carlson, John J. LiPuma

**Affiliations:** 1 Department of Molecular, Cellular and Biomedical Sciences, University of New Hampshire, Durham, New Hampshire, United States of America; 2 Department of Pediatrics and Communicable Diseases, University of Michigan Medical School, Ann Arbor, Michigan, United States of America; University of Hyderabad, India

## Abstract

The nematode *Caenorhabditis elegans* may be killed by certain pathogenic bacteria and thus is a model organism for studying interactions between bacteria and animal hosts. However, growing nematodes on prey bacteria may influence their susceptibility to potential pathogens. A method of axenic nematode culture was developed to isolate and quantify interactions between *C. elegans* and potentially pathogenic strains of the *Burkholderia cepacia* complex. Studying these dynamics in liquid solution rather than on agar surfaces minimized nematode avoidance behavior and resolved more differences among isolates. Most isolates of *B. cenocepacia*, *B. ambifaria* and *B. cepacia* caused 60–80% mortality of nematodes after 7 days, whereas isolates of *B. multivorans* caused less mortality (<25%) and supported nematode reproduction. However, some *B. cenocepacia* isolates recovered from chronic infections were much less virulent (5–28% mortality). As predicted, prior diet altered the outcome of interactions between nematodes and bacteria. When given the choice between *Burkholderia* and *E. coli* as prey on agar, axenically raised nematodes initially preferred most lethal *Burkholderia* isolates to *E. coli* as a food source, but this was not the case for nematodes fed *E. coli*, which avoided toxic *Burkholderia*. This food preference was associated with the cell-free supernatant and thus secreted compounds likely mediated bacterial-nematode interactions. This model, which isolates interactions between bacteria and nematodes from the effects of prior feeding, demonstrates that bacteria can influence nematode behavior and their susceptibility to pathogens.

## Introduction

To understand why only some bacteria are prone to cause disease, it is important to study how they interact with the organisms that are most likely targeted by their offenses or defenses. Nematodes, perhaps the most abundant and diverse metazoans [1], are excellent candidates. Relationships between bacteria and nematodes span the continuum of parasitism to mutualism: for some nematodes, bacteria are the preferred food source; for others, bacteria are necessary partners for infesting eukaryotic hosts, and for others still, bacteria are pathogenic. However, the conditions that influence how nematodes sense and respond to bacteria are not well understood, so new methods that further this research are needed. More specifically, using nematodes as model hosts for understanding infection requires greater understanding of how nematode physiology and behavior can be affected by bacteria.

Over the past decade, the nematode *Caenorhabditis elegans* has become a well established model for studying pathogenicity of bacteria, beginning with *Pseudomonas aeruginosa* [2], [3] and including a wide range of other pathogens since [4], [5], [6], [7]. For these assays, *C. elegans* populations are typically fed a weakened strain of *Escherichia coli* on solid growth media and then moved to a separate agar plate on which a lawn of the test bacterium has been grown. However, such assays could unintentionally favor either bacteria or nematodes for several reasons, including: 1) the structured agar environment allows nematodes to avoid the test bacteria, 2) agar may localize and concentrate bacterial secretions more than a liquid mass-action environment and 3) the prior use of *E. coli* as a nutrient source could influence worm behavior and susceptibility to a different bacterial species.

Evidence for the ability of *C. elegans* to alter its feeding behavior and its susceptibility to infection is building[8]. Nematodes have been shown to use a sensitive olfactory system that leads to rapid learning and pathogen avoidance [9]. *C. elegans* is able to sense a variety of molecules associated with bacterial pathogens, including secreted surfactants [6], quorum-sensing molecules [10], and LPS [11]. In addition, *C. elegans* can be conditioned to prefer various bacteria based on their prior exposure, but this learning can be altered by prior feeding and experience [9]: starving nematodes are more discriminatory than well-fed worms [12].

In this study we used a defined liquid medium to raise nematodes for a novel liquid-based assay of virulence that avoids the potential complications of agar and prior feeding on a different bacterial species. *C. elegans* was grown axenically in a chemically defined nutrient medium before being transferred to minimal buffer containing a standardized density of bacteria in multiwell plates. The dynamics of this head-to-head competition were assayed over the following week using visual scores of nematode viability and by the optical density of the bacterial culture fraction as an estimate of bacterial growth, which correlated with total numbers of viable bacteria. By comparison, other assays of nematode virulence have been less able to measure concurrent bacterial growth.

We applied this model to better understand the variation in pathogenicity of strains within the *Burkholderia cepacia* complex (Bcc). This group of 17 species [13] is functionally diverse and forms beneficial and antagonistic relationships with a range of hosts [14], [15], [16]. Most notably, Bcc can cause life-threatening pulmonary disease in persons with cystic fibrosis (CF) [17], [18], [19]. Previous studies have described a range of interactions of Bcc with nematodes [20], [21], [22], [23], [24], but how these differences relate to bacterial genotype [25] and the physiology of either the bacterial or nematode populations remain unclear. Nematode virulence was thus quantified for a collection of Bcc strains using this new model and previous methods for comparison.

Studying the interactions between Bcc and nematodes in this manner enabled observation of both competitors over several days and thus increased resolution of subtle differences between strains. This method also demonstrated how nematode diet can affect behavior and susceptibility to potential pathogens: nematodes grown axenically were more susceptible to Bcc pathogens than those fed a standard *E. coli* diet and were more attracted to lawns of these strains on agar. This experimental method enhances the use of *C. elegans* as a model of pathogenesis and enables further study of the signals by which bacteria and nematodes may manipulate each other in their natural environment.

## Results

### Development of a Monoxenic Liquid Assay of Bacterial-Nematode Interactions

Our initial objective was to develop a method of studying isolated interactions between nematodes and bacteria that avoided the prior effects of growing nematodes on bacteria such as the standard laboratory diet of *E. coli*. One of the best media formulations for this purpose is *C. elegans* habitation and reproduction medium [26], or CeHR, which is a nearly defined medium developed to study the molecular requirements for nematode growth and reproduction [26]. *C. elegans* can be continuously propagated for weeks in this medium.

We used this medium to develop a novel liquid-based assay for nematode virulence that avoids the effects of recent feeding on a different bacterium and of the complications of an agar substrate, in which nematodes may burrow and become difficult to visualize. *C. elegans* strain N2 was grown axenically in CeHR, washed, and transferred to minimal buffer containing a standardized density of bacteria in multiwell plates. A complete protocol for this method is available (Supplementary Protocol S1). The dynamics of this head-to-head competition were assayed over the following week: nematode viability was quantified by visual counts under a dissecting microscope and bacterial growth was quantified by optical density, which correlated with viable cell counts (Figure S1). Nematodes (and bacteria, if at high density) were easily seen and counted in this liquid environment as nematodes tended to wriggle in place rather than swim in any one direction; at higher magnification, nematode digestive tracts could also be visualized to confirm infectious state. The liquid environment also improved detection of nematode behaviors including those associated with illness (e.g. sluggishness). One disadvantage of this method relates to its use of more complex culture methods that must remain aseptic, much as tissue culture systems require.

### Interactions Between Different Bcc Strains and *C. elegans* Are Diverse and May Favor Either Competitor

**Figure 1:**
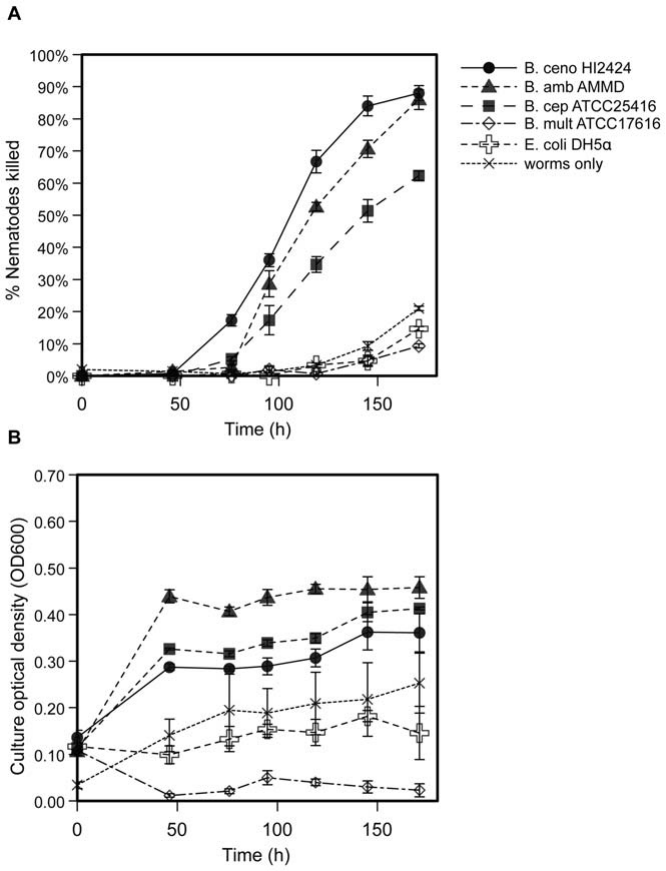
Relative virulence of representative strains of the *B. cepacia* complex. Strains vary significantly in their ability to kill *C. elegans* (mortality at 120 h, F = 264, p<0.0001). A. Percent nematode mortality over time. B. Density of planktonic fraction (OD600) over time. Results are expressed as mean (+/− SEM) of three replicate assays. B. ceno  =  *B.cenocepacia*; B. cep  =  *B.cepacia*; B. mult  =  *B. multivorans*; B. amb  =  *B. ambifaria*.

The outcomes of competitions between populations of *C. elegans*, a bacteriovore, and various Bcc species (Table 1) ranged from rapid nematode death to robust nematode growth (Figure 1, Table 2). Some strains, such as *B. multivorans* ATCC17616, supported nematode reproduction at the same rate or greater than those fed *E. coli*. For such avirulent strains, the optical density and colony forming units (cfu) of the bacterial fraction fell rapidly as nematodes grew. Strains moderately virulent to nematodes (e.g. *B. cepacia* ATCC25416) increased between two-and four-fold in density and suppressed nematode reproduction; these strains also caused many nematodes to become sluggish and resulted in 30–70% mortality. Strains highly virulent to nematodes (e.g. *B. cenocepacia* HI2424) grew similarly as moderately virulent strains but killed nematodes more quickly and to a greater extent (>60% mortality), with the onset of nematode death as early as 48 h and with the greatest increase in killing rate between 72 h and 96 h. In general, strains of *B. cenocepacia* and *B. ambifaria* were more virulent whereas strains of *B. cepacia*, *B. multivorans*, and *B. dolosa* were less virulent (9.3±0.6% mortality).

**Table 1:** *B. cepacia* complex species designations, strains used, and sources of isolation^a^.

Species name	Strain name (source of isolation: CF = cystic fibrosis)
*B. cepacia*	ATCC25416 (soil); HI2284 (CF)
*B. multivorans*	ATCC17616 (soil); HI2240 (CF)
*B. cenocepacia*	HI2424 (soil); J2315 (CF); AU1054 (CF); AU1107 (CF, [30]); PC184 (CF, [61];
*B. dolosa*	AU0645 (CF)
*B. ambifaria*	AMMD (soil); HI2502 (CF)

a All Bcc isolates were obtained from the *Burkholderia cepacia* Reference Laboratory and Repository at the University of Michigan and are identified and described in [62], except as noted.

**Table 2:** Summary of potential virulence mechanisms of tested strains and their association with nematode killing.

Strain: a	B.ceno HI2424	B.ceno AU1054	B.ceno AU1107	B.ceno J2315	B. ceno PC184	B.cep ATCC 25416	B. cep HI2284	B.mul ATCC 17616	B. mul HI2240	B.amb AMMD	B. amb HI2502	*E. coli* OP50	P. aerug. PA14
Nematodes killed in liquid^b^	67±4%	58±2%	3±0%	75±3%	17±4%	35±2%	66±2%	2±1%	21±1%	53±1%	74±1%	5±2.6%	3±2.0%
Nematodes killed on PGS agar^c^	44±5%	10±0%	12±10%	43±2%	–	43±1%	23±3%	6±2%	–	70±6%	–	0±0%	7±1.4%
Nematodes killed by toxin production^d^	84±4%	50±5%	0±0%	80±6%	–	–	39±5%	0±0%	–	–	43±1%	0±0%	41±12%
Protease production^e^	3	0	1	2.5	–	0	0	0	–	4	2.5	0	–
Swarming motility^f^	4.7±1.2	0	5.0±0.0	9.5±0.4	0	0	–	0	–	0	0	0	–
C8-AHL production^g^	5.18±0.18	18.37±0.76	5.16±0.42	6.54±0.46	2.04±0.11	18.52±0.36		1.90±0.03		18.98±0.38	5.57±0.34	1	1.33±0.03
Supernatant attraction^h^	3.55±0.63	1.0±0.51	7.25±1.46	4.44±1.37	–	2.63±0.52	–	1.04±0.23	–	0.97±0.41	–	1	1.08±0.01

a. B. ceno  =  *B.cenocepacia*; B. cep  =  *B.cepacia*; B. mult  =  *B. multivorans*; B. amb  =  *B. ambifaria*, P. aerug  =  *P. aeruginosa.*

b. Liquid killing  =  mean percentage of nematodes killed by ∼120 h, ± SEM (n = 3). *Burkholderia* strains vary significantly in lethality, F = 155.3, p<0.0001.

c. PGS agar killing  =  mean percentage of nematodes killed by 48 h on high-osmolarity PGS agar; ± SEM, (n = 3) --  =  not determined. *Burkholderia* strains vary significantly in lethality, F = 36.57 p<0.0001.

d. Toxin-mediated killing  =  mean percentage of nematodes killed by a toxin secreted by the test strain after 24 h; the toxin traversed a filter and was localized in agar. (± SEM, n = 3). nd  =  not determined. *Burkholderia* strains vary significantly in lethality, F = 129.2 p<0.0001.

e. Protease production  =  mm of clearing beyond colony growth after 40 h on milk agar. Readings were identical from three replicates, which prevents analysis of variance.

f. Swarming motility  =  mean mm of surface migration beyond the dense zone of swimming motility on 0.35% T-Soy agar after 24 h, (± SEM, n = 3). Motility varies significantly among *Burkholderia* strains: F = 53.8, p<.0001.

g. C8-HSL production  =  GFP emission at 514 nm following excitation at 475 nm of pAS-C8 in *P. putida* F117, scaled for OD600 of the log-phase Bcc culture prior to extracting its supernatant. Numbers are fold-increases over *E. coli* OP50, the negative control (mean ± SE, n = 6). Readings varied significantly among *Burkholderia* strains: F = 338, p<0.0001.

h. Supernatant attraction  =  ratio of worms (mean ± SE, n = 3) found near droplet of supernatant of test strain relative to that of *E. coli* following 1 h of incubation. Supernatants from different strains varied in their attractiveness, F = 7.37, p = 0.001.

**Figure 2:**
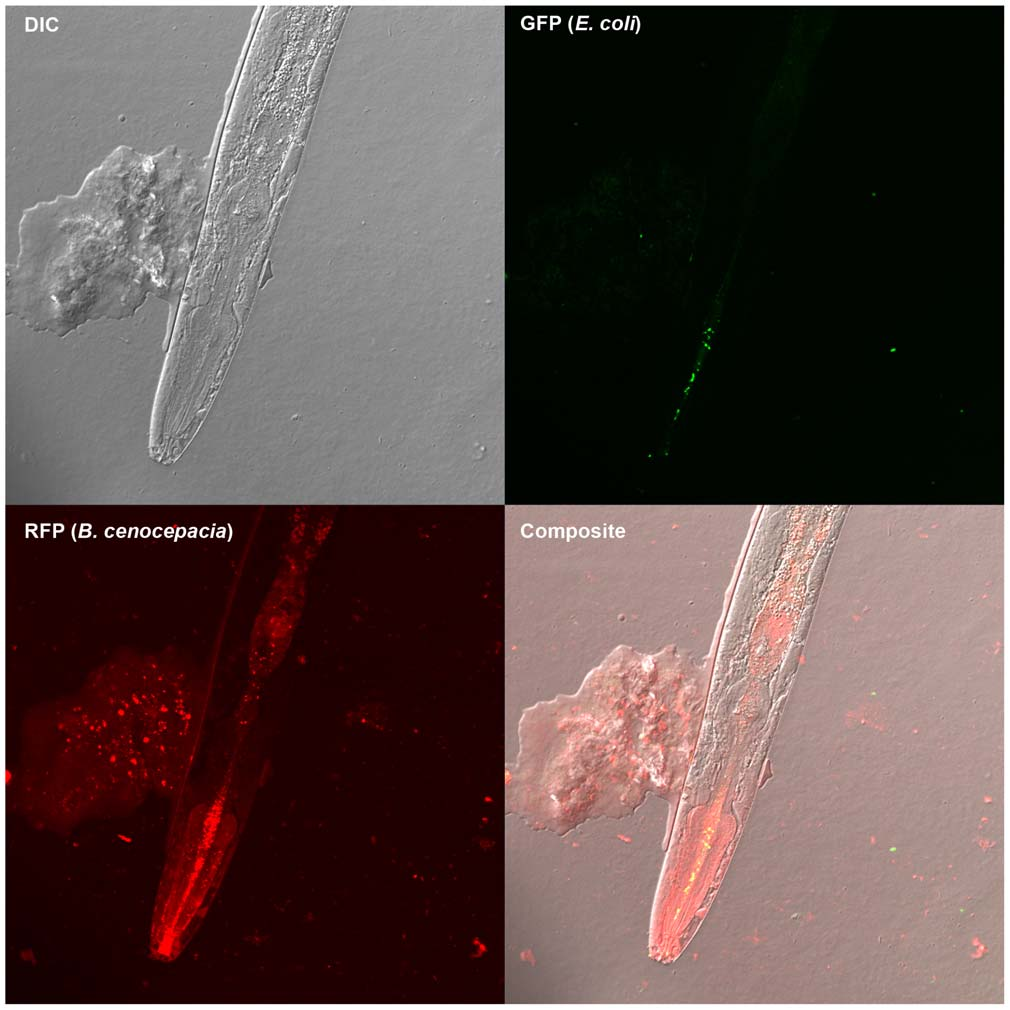
Localization of bacteria ingested by *C. elegans*. Confocal microscopy of a live nematode that was co-cultured with *E. coli* DH5α, marked with green fluorescent protein, and *B. cenocepacia* HI2424, marked with red fluorescent protein, is shown. *B. cenocepacia* (red, lower left quadrant) grows throughout the nematode gut and forms aggregates on the nematode cuticle, whereas *E. coli* (green, upper right quadrant) is found only in limited concentration in the mouth.

Nematodes succumbed to infections by virulent strains as bacteria accumulated throughout the gut at high density and in some cases formed aggregates on the nematodes (Figure 2). Following death, nematode corpses degraded more rapidly in cultures containing bacteria than in sterile culture and microscopy of nematode fragments revealed bacteria adhering to dead nematodes. These findings, combined with the fact that bacteria and nematodes are the sole source of carbon for each other in the assay, demonstrates that some *Burkholderia* can grow on dead nematodes. As controls for each assay, interactions with *E. coli* strains OP50 and DH5alpha, common food sources for laboratory cultures of *C. elegans*, and *P. aeruginosa* PA14, a strain known to kill nematodes under other conditions [27], [28], were measured. *E. coli* consistently supported robust nematode reproduction whereas *P. aeruginosa* was a lower quality food source, and neither strain noticeably grew nor killed nematodes (Figure 1, Table 2). We also included nematodes challenged by no bacteria in each assay as a negative control. In these wells modest changes in optical density of the culture occurred due to nematode death by other causes (often cannibalism). Cultures containing bacteria and nematodes that exhibited lower optical density than the bacteria-free controls were associated with the most active feeding of nematodes on bacteria (e.g. *B. multivorans* ATCC17616, Figure 1).

We also identified wide variation in nematode virulence among different *B. cenocepacia* genotypes (Table 2), many of whose genomes are completely sequenced (http://img.jgi.doe.gov) owing to their association with epidemic disease and broad metabolic potential. One of the most rapidly lethal is a soil isolate (HI2424 [29]) of the PHDC lineage, which was first characterized as the cause of an epidemic in the mid-Atlantic region of the U.S.[29]. PHDC strains have been defined on the basis of common genotyping profiles by several methods, including BOX-PCR[30] and multi-locus sequence typing [31], [32]. On the other hand, one of the isolates that best supported worm reproduction (strain AU1107, Table 2) was also a PHDC strain and was recovered from a chronically infected patient. We also assessed the pathogenicity of representative clones from two other epidemic lineages: strain PC184 from the Midwest epidemic strain type [33] produced low virulence, whereas strain J2315, a representative isolate of the ET12 epidemic strain type [34], was one of the most pathogenic.

### Comparison of Assays in Liquid Co-Culture with Assays on Agar

We conducted a different set of nematode virulence assays by plating strains on either fast-killing (PGS) or slow-killing (NGM) agar [2], [27] to compare with our findings from the liquid model. Only the most lethal strains (e.g. *B. cenocepacia* HI2424 and *B. ambifaria* AMMD) killed effectively on NGM agar; more often than not, worms ‘disappeared,’ presumably either burrowing into the agar or dying buried in the bacterial lawn. Our comparison thus focused on PGS assays, which produce more rapid mortality and are thus more easily scored (Table 2). Our results were generally consistent with previously reported effects of Bcc strains on *C. elegans* [20], [21], [24] and were usually qualitatively consistent with assays in our liquid model (linear regression of [mean % mortality on PGS agar, 24 h] versus [mean nematode virulence index in liquid, 119 h], r^2^ = 0.65, F_1,8_ = 11.0, p = 0.016). However, the rank order among strain virulence measures in liquid assays sometimes differed from those of solid assays. For example, *B. cenocepacia* strain AU1054 was proportionately less virulent on PGS agar than in liquid in comparison to other strains (Table 2). Further, the pathogenicity of several isolates (*B. cenocepacia* strains HI2424, AU1054, J2315) was much greater in our liquid model than either in our PGS agar assays or those conducted by others [20], [25].

### Toxin Production by Bcc Affects Nematode Survival

Variation in the pathogenicity of Bcc strains in our liquid model may be associated with differences in toxin production, among other mechanisms [21], [23], [35]. We reasoned that such toxins could pass through a filter, become trapped in agar, and potentially affect nematode survival even when bacteria are removed from the agar surface [27]. We found that the strains most pathogenic in our liquid model, *B. cenocepacia* strains HI2424, AU1054, and J2315 and *B. ambifaria* strains AMMD and HI2502, were also able to kill axenically-grown *C. elegans* in this manner (Table 2). As a positive control, *P. aeruginosa* PA14, known to secrete multiple toxins [27], [36], also killed rapidly in these assays. In contrast, other Bcc species, such as *B. dolosa, B. cepacia*, and *B. multivorans*, did not produce a toxin lethal to nematodes. The pathogenicity of the filtrates from different strains varied after 24 h of exposure to the worms (Table 2, ANOVA, F_1,7_ = 9.31, P<0.0001) but not after 4 h (not shown, ANOVA, F_1,7_ = 1.44, P = .264). We also evaluated whether Bcc must be alive and growing to kill nematodes or if their consumption alone is toxic. Though nematodes fed heat-killed HI2424 did not reproduce or appear as active as those fed *E. coli*, they did not succumb to this challenge (2.0% mortality ±0.6%). Prolonged UV exposure was surprisingly unreliable in killing HI2424 so we were unable to determine whether heat may have compromised the toxin.

The nature of this toxin or toxins remains unknown. We repeatedly tried and failed to isolate a toxic fraction from culture supernatants, perhaps because the toxin bound to the filters or because the toxin loses potency when dissolved in the supernatant. However, these results imply that one of the many secretion systems present in *B. cenocepacia* and *B. ambifaria* may cause nematode killing, although a mutant of the Type IV secretion system of *B. cenocepacia* K56-2 [37] had no effect on the moderately pathogenic parent strain (K56-2: 21%±6% mortality, K56-2Δptw: 28%±3% mortality). Because most Bcc have multiple secretion systems but only certain species and strains kill nematodes, differences in pathogenicity may depend on the composition of their secretions and how nematodes sense them.

### Prior Nematode Diet Affects Bacterial-Nematode Interactions

**Figure 3:**
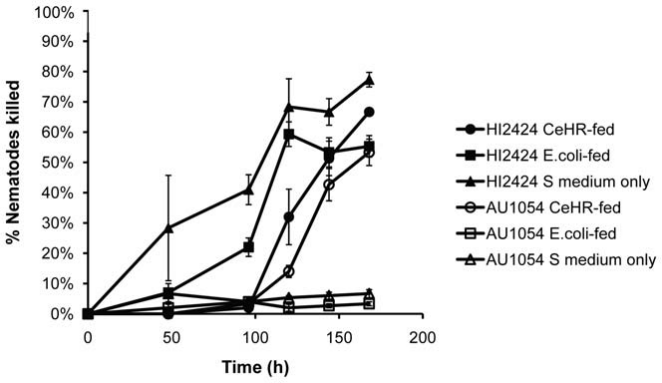
Effect of prior diet on susceptibility. *C. elegans* diet prior to encountering *B. cenocepacia* strains affects its susceptibility to killing. Strain HI2424 (filled symbols) is more lethal to nematodes that had been starved in buffer (triangles) or fed heat-killed *E. coli* OP50 (squares) than to nematodes that were grown in axenic CeHR medium (circles); strain AU1054 (open symbols) is less lethal to nematodes that had been fed *E. coli* or starved in buffer, than to nematodes that were grown in axenic CeHR medium. Results are expressed as mean (+/− SEM) of three replicate assays.

Bacterial isolates may vary in nematode virulence if they differ in palatability because in most cases bacteria must be consumed to be lethal. Nematodes may simply avoid feeding on pathogenic bacteria and persist in the test medium in dauer form. Further, nematode food preference may be influenced by prior feeding history [9], [12]. In our preliminary experiments using liquid nematode culture we noticed that nematodes fed *E. coli* appeared to differ in susceptibility than those grown axenically. To test the hypothesis that prior nematode diet influences susceptibility to infection, we challenged three sets of nematodes fed different diets with two closely related *B. cenocepacia* isolates of the PHDC clonal lineage, HI2424 and AU1054 [38]. These isolates are both virulent to nematodes but differ in their pathogenicity in liquid and by a larger margin on PGS agar (Table 2). The different nematode growth conditions were: i) hatched and matured in minimal S medium, which has little nutritive value and induces starvation and increased sensitivity to various food odors [9], ii) grown in S medium supplemented with heat-killed *E. coli*, or iii) grown in CeHR medium, a rich food source free of bacteria. Starving worms were most susceptible to killing by HI2424, which appeared able to kill and degrade nematodes entering dauer stage, but these nematodes were essentially resistant to AU1054 by avoiding its consumption (Figure 3, triangles). Similarly, worms fed killed *E. coli* were also killed by HI2424 but not AU1054 (Figure 3, squares). In contrast, CeHR-fed worms were killed at nearly identical rates by both isolates (Figure 3, circles). These results suggest that HI2424 may kill nematodes indiscriminately of their feeding behavior, but that AU1054 only kills feeding nematodes that have not experienced *E. coli* as prey. This led us to explore further how prior feeding experience of the nematodes influenced consumption of potential pathogens and susceptibility to infection.

**Figure 4:**
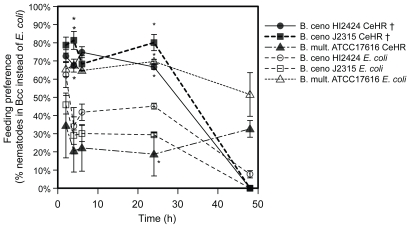
Effect of prior exposure to *E. coli* on nematode feeding preference. Nematodes raised in CeHR medium prefer lethal *B. cenocepacia* strains HI2424 and J2315 over *E. coli*, but the reverse is true for *B. multivorans* ATCC17616, which is nontoxic and is preferred by nematodes fed *E. coli*. †, pairings lethal to nematodes; other interactions supported nematode reproduction. Results are expressed as mean (+/− SEM) of three replicate assays. * p<0.05, 1 sample t-test versus expected 50%.

To isolate effects of prior diet on nematode feeding preference towards various Bcc isolates, we raised nematodes either in CeHR medium or in S medium supplemented with heat-killed *E. coli* and added each to agar plates containing a small lawn of *E. coli* on one side and one of several test Bcc strains on the other. Prior diet significantly influenced feeding preference for three strains (*B. cenocepacia* strains HI2424, J2315, and *B. multivorans* ATCC17616) (Figure 4, * p<0.05). CeHR-raised nematodes actually preferred the lethal *B. cenocepacia* strains HI2424 and J2315 over *E. coli* and this proved >50% fatal by 48 h. However, nematodes fed *E. coli* avoided these strains after a brief learning period and by 48 h the nematode populations grew on all plates. In contrast, nematodes fed *E. coli* preferred the avirulent strain *B. multivorans* ATCC17616 over *E. coli* as food, but CeHR-raised worms did not begin feeding on this strain until depleting the *E. coli* lawn (Figure 4). Feeding preference was not affected for *B. cepacia* ATCC25416, *B. cenocepacia* strain AU1054, or *B. ambifaria* AMMD (dynamics not shown, p>0.08 in all cases).

### Culture Supernatant of Some Strains Attracts Nematodes

**Figure 5:**
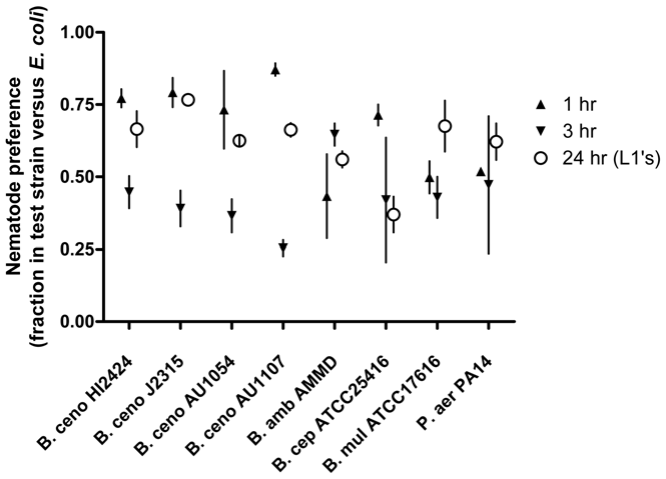
Nematode preference to bacterial culture supernatants. *C. elegans* prefers the spent cell-free culture supernatant of some Bcc strains to that of its prior *E. coli* diet. Cell-free supernatant from each Bcc strain was spotted opposite that from an *E. coli* OP50 culture. The fraction of nematodes traveling towards the Bcc supernatant within 1 h (up triangles) and then towards the *E. coli* supernatant by 3 h (down triangles) is shown. Within the first day, eggs from adult nematodes hatched and neonate migration at 24 h was observed (open circles). The error bars are SEM from three replicate assays.

To determine whether nematode preference was influenced by extracellular bacterial secretions, we quantified behavior of nematodes placed between cell-free supernatant of Bcc culture and of *E. coli* OP50 culture, using agar to localize these secretions. Nematodes initially preferred supernatant of most Bcc strains (Table 2, mean Bcc preference  = 66.6%, t = 2.93, p = 0.022) and most strongly for *B. cenocepacia* HI2424, J2315, and AU1107 (Figure 5). However, the nematodes soon left these areas by 3 h perhaps in search of other nutrition, in many cases returning to the *E. coli* supernatant. Supernatants were not lethal to the nematodes and their active, motile offspring that emerged 24 h later also preferred these strains, though less strongly than the adults on the previous day (Figure 5, mean Bcc preference  = 61.8%, t = 2.87, p = 0.024). Nematodes placed between two samples of *E. coli* supernatant did not differ significantly in nematode preference (n = 3, t = 0.92, p = 0.12), did not persist near these spots after 4 h, and preferred *E. coli* supernatant over an equivalent volume of T-Soy medium (20±3% nematodes near *E. coli* supernatant, 0% found near T-Soy at 24 h.). These results demonstrate that nematodes sense the secretions of their prey and chemicals alter nematode behavior.

In attempts to identify the physiological bases of how *Burkholderia* influences *C. elegans* feeding preference, we measured additional phenotypes that could relate to nematode chemoattraction for a subset of strains. Bacterial protease activity could release media components that are attractive, so we measured protease production of Bcc strains that vary in nematode preference (Table 2) but found no correlation between these traits. Next we quantified swimming and swarming motility on 0.35% agar since a surfactant molecule secreted by *Serratia* for motility has been shown to repel *C. elegans* [6], [9]. The *B. cenocepacia* strains HI2424, J2315, and AU1107 that attract naive nematodes also exhibited swarming motility on 0.35% agar; other strains did not produce this swarming ring. However, *B. cepacia* ATCC25416 was both attractive and nonmotile (Table 2). The quorum sensing molecules that are known to regulate both motility and protease production in Bcc, C8-homoserine lactones (C8-HSL) [39], are also potential nematode attractants[10]. We screened these same isolates for C8-HSL production using a fluorescent reporter strain and observed a range of activity from high (*B. cepacia* ATCC25416, *B. cenocepacia* AU1054, and *B. ambifaria* AMMD) to low (*B. multivorans* ATCC17616), but these values also did not correlate well with nematode preference (Table 2). The molecular bases of nematode feeding preference among these strains therefore remains unknown and may either result from a combination of these traits or by an distinct, unidentified mechanism. Future work will explore the genetic and phenotypic differences associated with changes in palatability and virulence.

## Discussion

Like most animals, nematodes are capable of pathogen avoidance behavior [40] and such behavior may be influenced by their recent feeding history [9], [12]. We found that the traditional method of growing *C. elegans* on its typical food source, *E. coli*, caused nematodes to avoid lethal bacteria more quickly and to a greater extent, which allowed more nematodes to survive. Thus, axenic nematode culture should be considered for future studies of bacterial pathogenicity because it tends to increase nematode susceptibility to virulent strains. Further, nematode sensitivity to the cues of pathogenicity appears to require training by prior exposure to non-pathogenic bacteria: naïve worms were attracted to the most virulent strains, whereas worms that had been fed bacteria were much more discriminating.

We evaluated several potential mechanisms that could underlie the positive association between pathogenicity and attractiveness of prey bacteria. Compounds secreted for motility, proteases, and quorum sensing are each potential attractants or repellents but none of these correlated well with the observed preference patterns (Table 2). Thus, the attractiveness of *Burkholderia* strains could differ by distinct combinations of these traits or perhaps as a result of substances we have not yet identified. Altering the metabolism of a few attractive and pathogenic bacterial strains by changing culture conditions or introducing a library of mutations could produce this evidence. Similarly, screening the behavior of selected *C. elegans* mutants with deficient or altered capacity for chemosensation [40] could aid this search and also shed light on the relationship between pathogen avoidance behavior and innate immune function [41]. The greater survival of nematodes that first fed on benign *E. coli* and then survived encounters with pathogenic *Burkholderia* could also result from upregulation of innate immunity by their initial prey. We suggest that our new method and findings facilitate further studies in this area.

A broader goal of these studies was to enhance the use of *C. elegans* as a model host for the study of pathogenesis in animals. For several years, *C. elegans* has been useful for the study of innate immunity and host-pathogen interactions [42] and has shown promise in demonstrating which mutations may affect pathogen susceptibility. However the greater promise of *C. elegans* as a model host may be the ability to screen many bacterial strains for subtle differences in virulence, given that nematodes are inexpensive and easily manipulated [4], [43]. Along these lines, effects of Bcc infections in humans are variable and these differences associate with the infecting strain type. For example, epidemiological tracking [44], [45], [46] and to a lesser extent patient outcome studies [47], [48], [49] suggest that *B. cenocepacia* infections are more threatening than *B. multivorans* infections.

It is therefore noteworthy that *B. multivorans* is essentially avirulent in this model host as well as in other hosts[50] and tends to be associated with more positive outcomes when it causes infection; in contrast, *B. cenocepacia* infections tend to be severe for both nematode and humans[47]. In addition, two *B. cenocepacia* isolates (AU1482 and AU1107) of the same strain type as the most virulent strain (HI2424, strain PHDC) but isolated from a longer-term, chronic infection were avirulent, which suggests that nematode pathogenicity may be lost during chronic infection or simply that these relatively avirulent strains could be more prone to cause long-term chronic infection. If the former explanation is true and chronic infection by Bcc is generally associated with a loss of pathogenicity traits, as it is for *P. aeruginosa* [51], [52], [53], then models such as this nematode system might be used to screen for these transitions. More generally, a better understanding of pathogenic variation among infecting Bcc strains will rely on the use of hosts such as nematodes that are naturally susceptible and likely encountered by these bacteria.

In summary, using axenically grown *C. elegans* as a model host to study bacterial infection is a promising extension of existing methods. By eliminating food bacteria from nematode culture we were able to demonstrate behavioral plasticity in nematode preference for different Bcc strains, with the most lethal strains paradoxically the most attractive. The products that underlie nematode attraction and killing are a subject of current study and if found may enable further tests of the hypothesis that bacterial pathogenicity mechanisms are broadly effective against metazoans [28], [50], [54].

## Materials and Methods

### Bacterial Strains, Media, Culture Conditions, and Phenotypic Assays

The origin and description of all Bcc strains used is described in Table 1. *P. aeruginosa* strain PA14 was a gift from G. O'Toole [55], *E. coli* OP50 [56] was a gift from C. Warren, and *E. coli* DH5α was from laboratory stock. Fluorescent marking was accomplished using pSPRed (Poltak and Cooper, submitted) and pHKT2 [57] by electroporation and positive selection on 50 µg/ml chloramphenicol. All strains were recovered from frozen stocks, streaked for single colonies on T-Soy Agar, and then propagated in Luria Broth or in T-Soy Broth without antibiotics at 37°C. Plating on *Burkholderia cepacia* selective agar (BCSA) [58] was used to rule out potential contaminants. Protease production was scored by measuring zones of inhibition on milk agar (T-Soy Agar +2% skim milk powder), and swimming and swarming motility were measured in mm of distance covered after 24 h incubation on 0.35% T-Soy Agar.

### Nematode Strains and Media

*C. elegans* strain N2 was obtained from the *Caenorhabditis* Genetic Center, Minneapolis, MN. Periodically, this strain was recovered from a frozen state and propagated in 1 L boxes of nematode growth medium (NGM) [56] +12.5 µg/ml nystatin seeded with *E. coli* OP50 as a food source. These conditions yielded >10^7^ worms after four days. Gravid adults from these boxes were then harvested by several rinses with M9 buffer, transferred to 25 ml conical tubes and pelleted by centrifugation for 3 min at 3000 rpm. The supernatant was discarded and the worms were resuspended in M9 to be centrifuged and washed twice more. In some assays, worms were instead purified by sucrose flotation [56].

### Nematode Virulence Assays

To synchronize nematode populations to a common age and stage, bleach was added to purified populations. The eggs within gravid adults are resistant to bleach and were isolated by centrifugation and added to M9 buffer, in which they hatched overnight. Hatched L1 larvae were added to 10 ml aliquots of *C. elegans* habitation and reproduction medium, or CeHR [59]. Briefly, CeHR is a complex and nearly defined medium consisting of salts, trace minerals and vitamins, nucleic acids, amino acids, glucose, and ultra high-temperature pasteurized skim milk. Its recipe can be found at http://usacehr.amedd.army.mil/cehr_medium.htm.

For virulence assays, samples of approximately 100 synchronized L4 worms that had been washed in M9 buffer were each placed in 3 wells of a 6-well plate with 5 ml S medium [56]. Following a wash in 1X phosphate-buffered saline, 500 µl samples of the bacterial population grown in LB for 18-24 h at 37°C and standardized to an OD_600_ of 1.0 were added to each well. The relationship between OD600 and colony forming units (cfu) was quantified for each bacterial strain under study; at stationary phase, the cfu of all different strains varied less than fivefold (data not shown). Assay plates were incubated at 22°C for up to nine days. Percent worm death was monitored by counting the number of dead worms per 50 total worms found in a contiguous area under a dissecting scope. Bacterial density was quantified following a 10 min period of static refrigeration to cause live nematodes, corpses, and fragments to sediment, and then by OD_600_ of an aliquot of the planktonic bacterial fraction in a cuvette. Absence of nematode fragments was verified microscopically in these aliquots. Each assay was typically conducted with threefold replication and then repeated entirely as a complete block to verify patterns. Rank order of bacterial strain virulence rarely varied between blocks, but the overall worm susceptibility varied among assays.

Assays of bacterial virulence to nematodes on solid agar were conducted as previously described [2], [27]. Briefly, overnight cultures of each strain were grown in LB, spread in a circle in the center of the agar plate, and grown for 24 h at 37°C. Either NGM agar or phosphate-glucose-sorbitol (PGS) agar, a richer, high osmolarity medium designed to enhance effects of secreted toxins [2], was used. A known number of synchronized L4 larvae, between 20–50, were added manually to the plate. Virulence was tracked by counting live and dead worms. Most death occurred by 48 h on PGS and by 96 h on NGM.

### Assays of Nematode Food Preference

Food preference was scored by inoculating 100 mm NGM agar plates with 30 µl of overnight culture from the test strain on one side and with the same volume of *E. coli* OP50 on the other side. Approximately 50 worms were added to the center of the plate. Worm behavior was tracked by counting worms near the spot locations at regular intervals beginning at 2 h. Each preference assay was replicated three times and scored as the percentage of nematodes found in or adjacent to the test strain relative to the number of nematodes in or adjacent to *E. coli*. Preference for secreted compounds was measured by isolating cell-free supernatant from overnight cultures by centrifugation and inoculating 30 ul on either side of a 100 mm plate. Approximately 50 worms were added to the center of the plate and their number and location counted at 1, 3, and 24 h.

### Bacterial Toxin Production Assay

Toxin production was assayed by adding 100 µl of overnight LB cultures to sterile 0.22 µm filter paper on the surface of PGS agar plates, followed by 20 h of incubation at 32°C. Filters were then removed and 5 µl (or ∼25 worms) of synchronized L4 stage *C. elegans*, grown previously in CeHR medium and washed in M9 buffer, were added to the center of agar. Worm viability was scored at 4 h and 24 h.

### Microscopy

To visualize how *Burkholderia* pathogens and *E. coli* prey bacterial populations within nematodes, confocal laser scanning microscopy was performed on mounts of *C. elegans* nematodes infected with single clones of bacteria at 200× magnification on a Zeiss LSM510 Meta. *Burkholderia* bacteria were marked with pSPRed (S. Poltak and V.S. Cooper, submitted) prior to infection of nematodes using mating procedures previously described [60]; these were visualized by excitation at 543 nm and emission through a BP560–615 nm filter. *E. coli* DH5alpha containing pSPY (Poltak and Cooper, submitted) was used as a control diet and was visualized by excitation at 488 nm and emission through a BP505–530 nm filter. Images were viewed and exported without manipulation using the Zeiss Zen Light software.

## Supporting Information

Figure S1 Correlation between optical density (OD600) and culturable bacteria (cfu/ml). Co-cultures of Bcc strains and nematodes show strong, positive correlation between OD600 and cfu/ml for most virulent strains (B. cep ATCC25416, r^2^ = 0.68; B.ceno HI2424, r^2^ = 0.51, B.ceno J2315, r^2^ = 0.50), generally positive but variable correlation for B. ceno AU1054 (r^2^ = 0.18), and significant correlation over limited ranges for avirulent strains because nematode feeding limits density (B. mul ATCC17616, r^2^ = 0.24, *E. coli* OP50, r^2^ = 0.52). Lines are derived from linear regressions of OD600 to cfu/ml.
(0.35 MB TIF)

Supplementary Protocol S1 Protocol: Axenic nematode culture for monoxenic assays of bacterial virulence.
(0.04 MB DOC)
