# Supplementary figures and images for: Susceptibility of Caenorhabditis elegans to Burkholderia Infection Depends on Prior Diet and Secreted Bacterial Attractants
Source: PLoS One. 2009 Nov 23;4(11):e7961. doi: 10.1371/journal.pone.0007961 (PMC2776534; doi:10.1371/journal.pone.0007961)

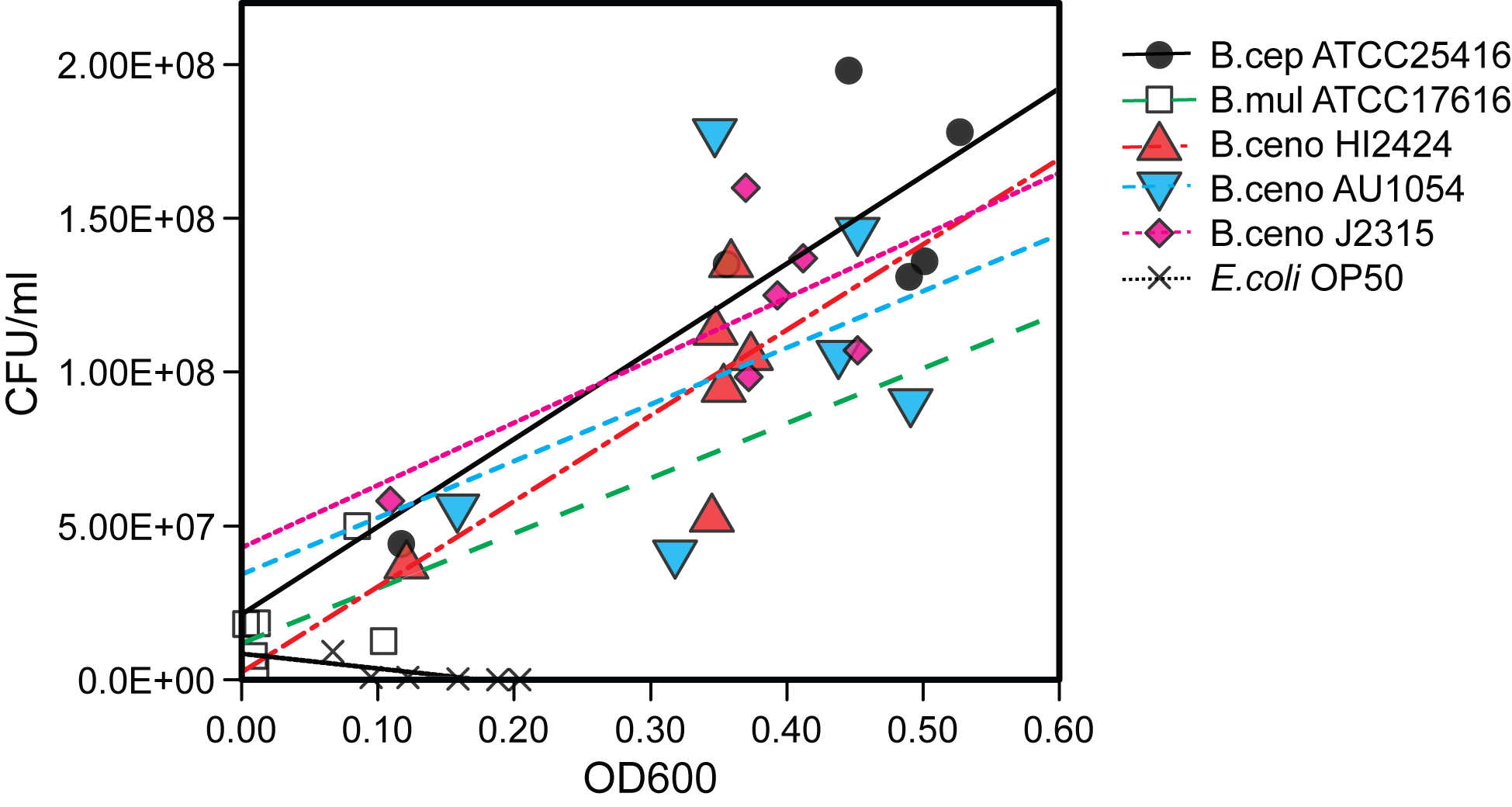

Supplement: Figure S1 — Correlation between optical density (OD600) and culturable bacteria (cfu/ml). Co-cultures of Bcc strains and nematodes show strong, positive correlation between OD600 and cfu/ml for most virulent strains (B. cep ATCC25416, r2 = 0.68; B.ceno HI2424, r2 = 0.51, B.ceno J2315, r2 = 0.50), generally positive but variable correlation for B. ceno AU1054 (r2 = 0.18), and significant correlation over limited ranges for avirulent strains because nematode feeding limits density (B. mul ATCC17616, r2 = 0.24, E. coli OP50, r2 = 0.52). Lines are derived from linear regressions of OD600 to cfu/ml. (0.35 MB TIF) [file pone.0007961.s001.tif]
