# Supplementary material for: Susceptibility of Caenorhabditis elegans to Burkholderia Infection Depends on Prior Diet and Secreted Bacterial Attractants
Source: PLoS One. 2009 Nov 23;4(11):e7961. doi: 10.1371/journal.pone.0007961 (PMC2776534; doi:10.1371/journal.pone.0007961)
Supplement: Supplementary Protocol S1 — Protocol: Axenic nematode culture for monoxenic assays of bacterial virulence. (0.04 MB DOC) [file pone.0007961.s002.doc]

**Protocol: Axenic nematode culture for monoxenic assays of bacterial virulence**

**Step 1: Nematode growth**

1. **Harvesting**: Collect worms from a large pipet box containing 0.5L of nematode growth medium[1]. Nematodes should be densely populated but not starved (seeded with *E. coli* OP50 five days prior and worms added to the culture 4 days prior). To harvest, hold the box at an angle and pipet 10ml of room temp M9 buffer down the surface of the agar. Collect the fluid at the bottom. Continue to rinse the same M9 down the box 8 – 10 times more. Pipet up the fluid and place it in a labeled 50ml conical.
2. Repeat this process again with a new pipet and another 10ml of M9 buffer. Add this second worm solution to the first collection in the conical.
3. **Washing**: Centrifuge the worm solution at 3000rpm at 22°C for 3 minutes with a counterbalance to pellet the worms. Carefully pour off the supernatant.
4. Wash by adding 4ml of cold M9 buffer and mix to resuspend, then pellet at 3000rpm for 3 minutes. Pour off the supernatant.
5. Wash by adding 10ml cold M9 buffer and mix to resuspend then pellet at 3000rpm for 3 minutes. Pour off the supernatant.
6. **Sucrose float**: Add in layers to the washed worms: 5ml of cold M9 buffer (gently mix to resuspend the worms), 5ml of cold 60% sucrose solution, then 2ml of cold M9 buffer. Do not mix after the sucrose or second layer of M9 has been added.
7. Centrifuge at 3000rpm for 5 minutes. Live worms will be at the interface of the M9 and the sucrose.
8. Transfer the upper layer to a new labeled 50ml conical being very careful to get the layer of live worms without pipetting up the sucrose.
9. If all the worms were not collected, add 1-3ml of cold M9 buffer to the top of the remaining sucrose to assist in collecting the remaining worms. Add the second collection of worms to the first in the new conical. (Dilute down with M9 buffer if sucrose was taken up with the worms or they won’t pellet).
10. Centrifuge at 3000rpm for 3 minutes, pour off the supernatant.
11. Wash by adding 10ml of room temperature M9 buffer, mix to resuspend and pellet at 3000rpm for 3 minutes.

**Step 2: Preparation of sterile *C. elegans* embryos for liquid culture**

1. Follow the SOP for “Cleaning Worms for Egg Prep”. After step 11, pour off the supernatant from the cleaned worm pellet leaving about 1ml of the M9 buffer with the washed worms. Mix briefly to resuspend the worms.
2. Prepare bleach mix: 10ml bleach (in black refrigerator)

2.5ml 10N NaOH (in black refrigerator)

12.5ml H2O.

1. Add the 25ml of ice cold bleach mix to the 1ml of worms in the conical.
2. Bleach approximately 5-10 minutes, vortex mixing periodically. Watch under the microscope to see when the worms actually open up to release the eggs.
3. As soon as the eggs have been released, fill the conical to the top with room temperature M9 buffer to dilute the bleach.
4. Centrifuge at 3000rpm for 3 minutes to pellet the worms.
5. Pour off the supernatant. Add 50ml of the room temp M9 buffer, mix, centrifuge at 3000rpm for 3 minutes.
6. Repeat above step two more times to thoroughly wash the eggs (total of 3x50ml washes).
7. After third wash, pour off all but 1-2ml of the supernatant and mix to resuspend the eggs.
8. Pipet the eggs in M9 into a culture flask containing 10ml of M9 buffer. Place the cap on tightly then back it off ¼ turn to allow in a slight amount of air.
9. Clean the entire outside of the flask with 70% ethanol and place it on the platform shaker at 62rpm in the 22 C incubator to hatch overnight.

**Step 3: Transfer hatched L1 larvae to CeHR liquid medim**

1. Check under microscope to make sure that the eggs have hatched to L1s.
2. With careful attention to sterile technique, remove all worms from the culture flask with a flamed 10ml pipet and transfer them to a 50ml labeled conical.
3. Centrifuge with a counterbalance at 4°C at 3000rpm for 5 minutes.
4. Carefully draw off the supernatant with a pipet and discard, leaving 1-2ml of the M9 worm solution.
5. Gently shake to resuspend the worms.
6. Using the hand pipet, measure how much worm solution is left and divide that into two new culture flasks, each containing 8ml of CeHR (<http://www.lib.umd.edu/drum/bitstream/1903/2204/1/CeHR_medium.htm>) (2)
   and 2ml of the UHT milk (from the -80 C freezer).
7. Tighten the two caps completely, then back off ¼ turn to allow in a slight amount of air. Clean the entire outside of the flasks with 70% ethanol, then place them in the 22 C incubator on the platform shaker at 62rpm for three days.

**References**:

1. Sulston J, Hodgkin J (1988) Methods. In: Wood WB, editor. The Nematode Caenorhabditis elegans: Cold Spring Harbor Press. pp. 587-606.

2. Rao AU, Carta LK, Lesuisse E, Hamza I (2005) Lack of heme synthesis in a free-living eukaryote. Proc Natl Acad Sci U S A 102: 4270-4275.
